# Asthma diagnosis and treatment – 1009. A clinical study of NE-C900 (OMRON) nebulizer

**DOI:** 10.1186/1939-4551-6-S1-P9

**Published:** 2013-04-23

**Authors:** TU Sukumaran, Ruby Pawankar, Jose Ouseph

**Affiliations:** 1 Dept.Of Paediatrics, Pims Tiruvalla, Kerala, Kerla, India; 2 Allergy and Rhinology, Nippon Medical School, Tokyo, Japan

## Background

Jet nebulizer systems that are used to generate therapeutic aerosols vary in their bronchodilating effect. The purpose of the present study was to compare the bronchodilator response in children with asthma between NE–C900 (OMRON), a new jet nebulizer and a commercially available nebulizer Redimist (RE).

## Methods

Asthmatic children who have consented to the study and aged 7 to 13 years with less than 70% of predicted PEFR were randomized in to 2 groups (30 subjects in each group). One group received Salbutamol solution (0.15mg of Salbutamol nebulizer solution / kg body weight diluted in 2ml of saline solution) nebulized by NE–C900 (OM). The other group received. Salbutamol solution nebulized by Redimist. At least 3 maneuvers were done to .obtain the most acceptable PEFR before Salbutamol inhalation and at 15 and 30 minutes after inhalation. Statistical analysis was done using the ANOVA method to analyze the changes in lung function at 15 and 30 min, Probability (p) values of less than 0.05 was considered to indicate a statistically significant difference.

## Results

There was no difference in PEFR at baseline between both groups. The difference in PEFR between that at baseline and that at 15 minutes, as well as between that at baseline and that at 30 minutes were highly significant for both OM and RE groups. The improvement in PEFR value for OM group was greater as compared to that of the RE group at 15 min and the difference was statistically significant at p = 0.005. But the difference in the improvement in PEFR between that at 15 minutes and that at 30 minutes for both groupswas not statistically significant. Comparing the variances of repeatedly measured PEFR values, ANOVA showed that there was consistency in the data and no significant differences in variances were detected in both groups at baseline, 15 minute or at 30 minute.

## Conclusions

This study clearly shows that the bronchodilating effect as reflected by the improvement in PEFR value at 15 minutes was greater for NE–C900 as compared to Redimist.

